# From Data to Dose: Diversity in Therapeutic Drug Monitoring and Pharmacokinetics-Based Individualization of Drug Therapy

**DOI:** 10.3390/pharmaceutics17081083

**Published:** 2025-08-21

**Authors:** Gellert Balazs Karvaly, Barna Vásárhelyi

**Affiliations:** Department of Laboratory Medicine, Faculty of Medicine, Semmelweis University, 4 Nagyvárad Square, H-1089 Budapest, Hungary; vasarhelyi.barna@semmelweis.hu

## 1. Introduction

In the past few years, interest in therapeutic drug monitoring (TDM) and the pharmacokinetics-based individualization of drug therapy has gained increased impetus. A recent search of the database of the National Library of Medicine, National Center for Biotechnology Information (Bethesda, MD, USA, https://pubmed.ncbi.nlm.nih.gov, accessed on 6 August 2025) yielded a more than twofold growth in the number of hits from 2022 to 2025 for the search term “model-informed precision dosing”, while the number of hits for the search term “population pharmacokinetic modeling” has remained steadily at around 1200 since 2020. These models have begun to be implemented in real-life clinical settings as an increasing number of clinical laboratories are gaining access to the technologies required for conducting therapeutic drug monitoring, new generations of pharmacometricians are emerging, and the use of artificial intelligence is rapidly becoming widespread [1,2,3,4,5]. New goals have appeared on the horizon such as continuous drug level monitoring using biosensors, the use of saliva measurements to gain information on drug exposure, the use of machine learning to select the best-performing model for a specific situation from a model library, and the generation of AI agents [6,7,8,9]. The transformation of pharmacokinetics into a multidisciplinary 21st-century field of medicine leveraging state-of-the-art technologies, with an established impact on clinical decision making, is in progress.

This Special Issue, “Therapeutic drug monitoring and pharmacokinetics-based individualization of drug therapy, 2nd edition”, is a continuation of the first collection launched in 2021. Being open for submissions at exactly the same moment as this paradigm shift is taking place, the two compilations allow readers to obtain a detailed overview of the switch in focus from measuring single samples and comparing drug assay results to a therapeutic range, to implementing carefully designed dosing and sampling regimens, and using sophisticated mixed-effects modeling software to construct population-level and individual pharmacokinetic models for the benefit of each patient.

## 2. Overview of Published Work

The administration of the drugs investigated in the research articles published in this Special Issue requires special attention. In order to evaluate exposure, sensitive, accurate, and reproducible assays must be used. Recently, a new approach to sample preparation, consisting of the use of magnetic microbeads, has emerged. A novel liquid chromatography–tandem mass spectrometry method relying on magnetic bead extraction is presented in Contribution (1) for the simultaneous quantification of the intracellular concentrations of two widely used immunosuppressants, tacrolimus and mycophenolic acid, in peripheral blood mononuclear cells (PBMCs) isolated from the blood samples of renal transplant recipients. PBMCs are the targets of these substances; therefore, the method enables pharmacokinetic monitoring that goes beyond traditional plasma measurements by effectively quantifying intracellular exposure and offering insights into variability in target-cell drug accumulation.

While effective sampling and analysis are crucial for assessing exposure properly, another important aspect of individualized drug administration is the carefully designed tailoring of dosage regimens based on the evaluation of drug assay results. A population pharmacokinetic model for the administration of tacrolimus as a meltdose formulation to elderly kidney transplant recipients is constructed by the authors of Contribution (2), and limited sampling strategies (LSSs) are assessed for TDM. LSSs that use two strategically timed post-dose samples are able to accurately approximate the full area under the concentration–time curve, supporting a reduction in patient burden without sacrificing accuracy. The meltdose formulation allows for a consistent PK behavior of the drug in this especially vulnerable patient population.

An emerging field of TDM is the monitoring of orally administered protein kinase inhibitors for oncological and related treatments. It is becoming increasingly apparent that, in a chemical, pharmacological, and biological sense, the substances belonging to this drug category exhibit considerable heterogeneity. A clinical algorithm is presented in Contribution (3) to integrate the plasma ponatinib trough concentration, molecular response status, and toxicity profile in order to personalize dosing in chronic myeloid leukemia patients. The approach presented emphasizes that dose reduction—if clinically indicated—does not necessarily compromise molecular outcomes, and may lower the burden of adverse events.

In Contribution (4), constitutional single-nucleotide polymorphisms that correlate with plasma imatinib trough concentrations are identified in chronic-phase chronic myeloid leukemia patients receiving standard imatinib therapy. Fifty SNPs show significant associations with imatinib trough concentration, and a subset corresponds to genes that are overexpressed in high-concentration patients. The findings underscore that host genetics—particularly variants affecting drug transit or cellular entry—contribute meaningfully to imatinib pharmacokinetics.

Biological therapy remains a rapidly emerging field of interest in TDM. Ustekinumab, a monoclonal antibody, has been in use for the treatment of inflammatory bowel disease since 2016. Contribution (5) discusses whether ustekinumab clearance predicts disease control more effectively than conventional trough serum concentrations. Lower ustekinumab clearance correlates more strongly with sustained disease control compared to trough levels. Clearance shows a stronger association with clinical remission and biomarker normalization. Trough levels are less predictive, especially during maintenance therapy. These findings suggest that clearance is a more reliable indicator of therapeutic adequacy.

Another example underscoring the utility of protein assays in TDM is the research detailed in Contribution (6), which introduces a multiplexed mass spectrometry–minimal residual disease (MS-MRD) methodology for simultaneously evaluating the concentration of M-protein and therapeutic CD-38-targeting antibodies (daratumumab, isatuxomab, talquetamab, teclistamab and tocilizumab), amended by the measurement of immunoglobulin levels using immunoturbidimetry, in multiple myeloma patients. The multiplexed MS-MRD assay quantifies M-protein clearance, the pharmacokinetics of therapeutic antibodies, and the recovery of normal immunoglobulin levels. This provides a detailed, time-resolved view of response kinetics, drug persistence, and immunosuppression. The method demonstrates potential for more nuanced monitoring in the treatment of multiple myeloma, helping to distinguish between therapeutic antibody presence and residual disease and guiding tailored treatment decisions.

TDM is clearly an important tool for managing psychiatry patients, considering the uniqueness of every case. Fluoxetine is an antidepressant that is employed widely for obsessive–compulsive disorder from seven years of age, and for major depressive disorder from eight years of age. The TDM-VIGIL trial, presented in Contribution (7), was conducted with the aim of investigating the relationships between dosage, serum concentration, and various predictors, and to provide an age- and indication-specific therapeutic range for fluoxetine and norfluoxetine in a naturalistic, multicenter prospective cohort of pediatric patients. A strong relationship between the fluoxetine dose and the serum levels of the fluoxetine active moiety was observed. The results of this carefully designed study can be useful for optimizing fluoxetine dosing regimens in the treatment of mood disorders by pediatric psychiatrists.

Finally, the authors of Contribution (8) argue for combining TDM with pharmacodynamic biomarkers to improve precision in antimicrobial dosing. They encourage the repurposing of diagnostic biomarkers, such as inflammatory markers or pathogen clearance metrics, as pharmacodynamic indicators could better reflect real-time treatment response. They also suggest that integrating these biomarkers with TDM data allows for more informed dosing decisions, potentially optimizing efficacy and minimizing toxicity.

## 3. Future Perspectives

While TDM and clinical pharmacokinetics are broad disciplines, the Guest Editors believe that three main topics will shape further progress. First, the steady progress made in well-defined areas, such as those represented by the publications appearing in this Special Issue, indicates that there is an increasing clinical demand for therapy individualization based on TDM and pharmacokinetic modeling, which, therefore, will certainly be part of a growing number of clinical decision making algorithms. Second, artificial intelligence bears enormous scientific and clinical potential, and has the capacity to radically shape the evolution of this field in the long term. Finally, novel bioanalytical technologies and methodologies that are now on the horizon may prove to be transformative in terms of the sampling site used and the quantity of data collected, as well as in their transmission to other software modules and their capacity to reduce assay turn-around times. With the increasing clinical acceptance of model-informed precision dosing, it will be truly exciting to see how the future is shaped by these novel tools.
